# The effect of 808 nm and 905 nm wavelength light on recovery after spinal cord injury

**DOI:** 10.1038/s41598-019-44141-2

**Published:** 2019-05-21

**Authors:** Barbora Svobodova, Anna Kloudova, Jiri Ruzicka, Ludmila Kajtmanova, Leos Navratil, Radek Sedlacek, Tomas Suchy, Meena Jhanwar-Uniyal, Pavla Jendelova, Lucia Machova Urdzikova

**Affiliations:** 1 Institute of Experimental Medicine, Academy of Sciences, Prague, Czech Republic; 2 2nd Faculty of Medicine, Charles University, Prague, Czech Republic; 3 3rd Faculty of Medicine, Charles University, Prague, Czech Republic; 4 Department of Health Care Disciplines and Population Protection, Faculty of Biomedical Engineering, Czech Technical University, Kladno, Czech Republic; 5 Laboratory of Biomechanics, Department of Mechanics, Biomechanics and Mechatronics, Faculty of Mechanical Engineering, Czech Technical University in Prague, Prague, Czech Republic; 6 New York Medical College, New York, USA

**Keywords:** Spinal cord injury, Spinal cord diseases

## Abstract

We investigated the effect of a Multiwave Locked System laser (with a simultaneous 808 nm continuous emission and 905 nm pulse emission) on the spinal cord after spinal cord injury (SCI) in rats. The functional recovery was measured by locomotor tests (BBB, Beam walking, MotoRater) and a sensitivity test (Plantar test). The locomotor tests showed a significant improvement of the locomotor functions of the rats after laser treatment from the first week following lesioning, compared to the controls. The laser treatment significantly diminished thermal hyperalgesia after SCI as measured by the Plantar test. The atrophy of the soleus muscle was reduced in the laser treated rats. The histopathological investigation showed a positive effect of the laser therapy on white and gray matter sparing. Our data suggests an upregulation of M2 macrophages in laser treated animals by the increasing number of double labeled CD68+/CD206+ cells in the cranial and central parts of the lesion, compared to the control animals. A shift in microglial/macrophage polarization was confirmed by gene expression analysis by significant mRNA downregulation of *Cd86* (marker of inflammatory M1), and non-significant upregulation of *Arg1* (marker of M2). These results demonstrated that the combination of 808 nm and 905 nm wavelength light is a promising non-invasive therapy for improving functional recovery and tissue sparing after SCI.

## Introduction

Traumatic spinal cord injury (SCI) is a debilitating condition with tremendous life-long consequences predominantly affecting young men. Patients’ lives are influenced by sensorimotor function deficits, dysfunction of the autonomic system and neuropathic pain; they also often develop subsequent complications including cardiovascular diseases, muscle atrophy and osteoporosis^1,2^. A mechanical trauma to the spinal cord can be induced by compression or contusion. The primary injury causes an immediate disruption to the cells and tissues, which is followed by a secondary cascade including neuronal apoptosis, glial cells activation, and inflammation. After the initial trauma, monocytes migrate from the blood stream to the injury site and the residual glia cells (microglia and astrocytes) are activated. Macrophages have a wide spectrum of different functions including promoting inflammation, phagocytosis, stimulating cell proliferation and releasing anti-inflammatory cytokines^3^. Their phenotype is influenced by the environment; many substances and/or surrounding cells can stimulate them^4^. The dual role of macrophages after central nervous system (CNS) injury is associated with a different kind of activation (polarization). The M1 macrophages (classically activated) and M2 (alternatively activated) macrophages^5^ are of interest and are being examined. M1 macrophages play a role in the inflammatory reaction; conversely M2 macrophages are usually described as anti-inflammatory cells. However, recent reports suggest that a strictly M1 or M2 macrophage phenotype is based mainly on *in vitro* experiments, and in organisms that can react to the surrounding environment by mixed phenotype^6^. Following SCI, most macrophages are polarized into M1 macrophages and only a small number of cells have M2 phenotype^7^. The low number of anti-inflammatory M2 macrophages likely contributes to the prolonged inflammatory response, and this may have negative effects on tissue preservation and axon regeneration^8^.

The initial primary injury also causes a mechanical disruption to the spinal cord vascular system, which results in vasoconstriction followed by hypoperfusion, ischemia, hemorrhage and edema^9–11^. Ischemia causes a reduction of oxygen delivery, which directly decreases the ability of mitochondria to maintain homeostasis^9,12^. Neurons are very dependent on mitochondrial metabolism and ATP production and the mitochondrial dysfunction after the CNS injury has been suggested as crucial for the subsequent neuronal cell death and the propagation of secondary injury^13–15^.

Photobiomodulation (PBM), also known as low level light therapy, represents the use of light to stimulate cellular functions to produce a therapeutic effect on living tissue. The hypothesis of how the PBM works is based on the fact that cytochrome c oxidase absorbs the light up to 920 nm^16^. This process could influence the binding of nitric oxide to cytochrome oxidase which blocks the cellular respiration. This photodissociation may reverse the signaling consequences of excessive nitric oxide binding^17,18^ and may explain the increase of the enzyme activity, oxygen consumption and ATP production after irradiation^19^. The effect of PBM is supported by results *in vitro* and *in vivo*. Karu *et al*.^20^ recounted the immediate effect of this in cultured cells.

PBM has been clinically used as an evidence-based approach in medicine^21–26^. Clinical studies indicate a potential therapeutic effect for traumatic brain injury^27–29^, stroke^30^, and neurodegenerative diseases^31,32^. There are a few studies investigating the effect of PBM after SCI^33–35^. Other studies have demonstrated the effectiveness of neural tissue regeneration by decreasing edema, preserving the tissue, influencing the chemical mediators involved in inflammation, and promoting functional recovery^33,36,37^.

In this study, the effect of PBM mediated by two different wavelengths (808 nm continuous and 905 nm pulsed), was tested in a rodent model of SCI. Multiwave Locked System (MLS) laser (ASA Srl, Italy) was used and has been clinically approved for safety. The controlled and synchronized energy should provide a more effective method of PBM therapy, enabling the distribution of energy over a wide area in a homogenous method. We evaluated functional recovery, soleus muscle mass, microglia/macrophage polarization and histopathological and molecular changes of the spinal cord after the compression of SCI.

## Results

### Experimental design

As a model of SCI, we used the previously described balloon-induced compression lesion to the spinal cord at thoracic level 8–9^38^. Following surgery, the rats were randomly distributed into two groups. The laser group received laser treatment applied to the injury site 15 minutes after the induction of spinal cord injury and then received this treatment for 10 consecutive days – once per day. The control group underwent the same treatment without application of the light therapy. The rats were checked 1 day after SCI and the animals with a BBB score higher than 2 were excluded from the experiment.

### The behavioral effect of photobiomodulation after SCI

**Figure 1:**
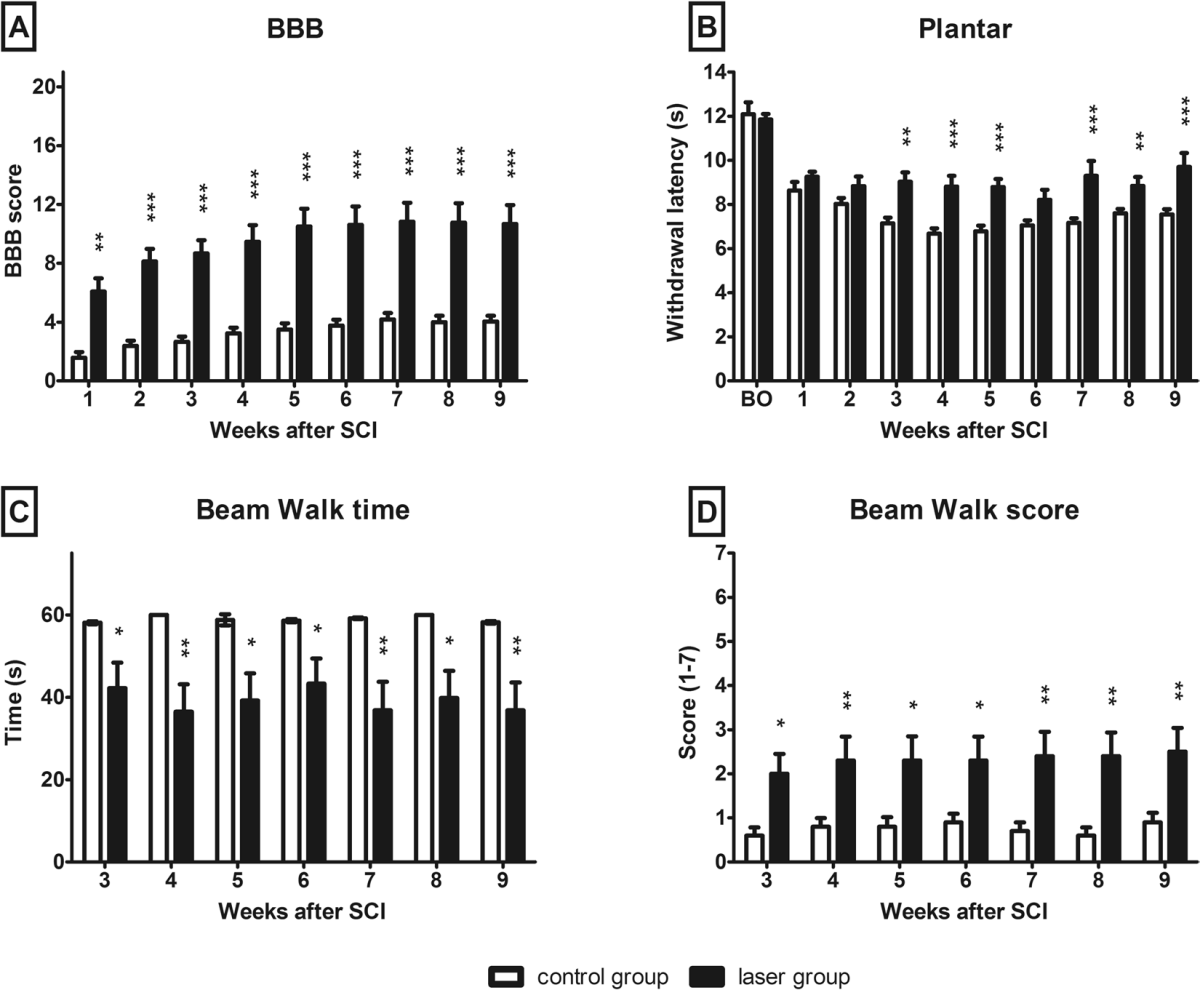
Functional recovery after SCI. (**A**) The assessment of locomotor activity in open field BBB test. Animals treated with laser showed a significantly faster locomotor recovery compared to the control group. (**B**) Plantar test for sensitivity to thermal stimulus. The hind paw withdrawal latencies of the laser-treated group were significantly higher compared to control animals and almost reached the pre-injury values. (**C**,**D**) Complex motor performance evaluated by beam walk test. Laser treated group showed better performance and needed less time to cross the beam. The values are expressed as mean ± SEM. Control group n = 11, laser-treated group n = 15. *p < 0.05, **p < 0.01, ***p < 0.001. BO – before operation.

Adult Wistar rats were tested by the Basso, Beattie and Bresnahan (BBB) test and the Plantar test weekly, from the first week following SCI. The results of the BBB test showed a significant increase of the scores immediately after the injury and continually from the first week (p < 0.01) to the 9^th^ week (p < 0.001) (Fig. 1A). The withdrawal latency of the hindlimbs to the thermal stimulus decreased after the SCI. Laser treatment reduced the post-injury hyperalgesia significantly in the 3^rd^ (p < 0.01), 4^th^ (p < 0.001), 5^th^ (p < 0.001), 7^th^ (p < 0.001), 8^th^ (p < 0.01) and 9^th^ (p < 0.001) week after the injury, as measured by the plantar test (Fig. 1B).

We used the Beam walk test to evaluate the recovery of coordination and motor function of the hindlimbs from the 3^rd^ week. The task was to cross the narrow beam within 60 seconds and the time required to pass the beam was then measured accordingly. The laser group displayed better test results than the control group (p < 0.05 in 3^rd^, 5^th^, 6^th^, 8^th^ week and p < 0.01 in 4^th^, 7^th^ and 9^th^ week). One of the eleven control animals was able to cross the beam as opposed to seven of the fifteen rats from the laser group (Fig. 1C). The animals were scored according to their locomotor coordination ability and balance throughout testing. The laser group achieved a higher beam walk score throughout the entire experiment (Fig. 1D, p < 0.05 in 3^rd^, 5^th^, 6^th^ week and p < 0.01 in 4^th^, 7^th^, 8^th^ and 9^th^ week).

### Kinematic analysis

**Figure 2:**
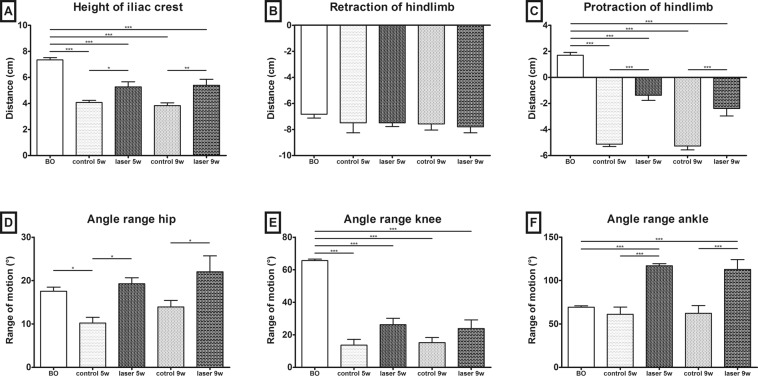
Locomotor recovery quantification. (**A**) The height of iliac crest defined as the vertical distance between the iliac crest and the runway. Both groups showed a reduced height of iliac crest after the SCI, compared to the healthy animals. Height of iliac crest was significantly increased in laser-treated animals 5 and 9 weeks after the SCI. (**B**) Maximal negative excursion (retraction) of hindlimb was not changed after the SCI. (**C**) Maximal positive excursion (protraction) was significantly decreased in all groups after SCI compared to healthy animals before operation. Laser-treated group showed significant improvement of protraction in both time points. (**D**) Significant decrease of the range of movement in control group compared to laser group. (**E**) Significant decrease of angle range in knee angle in both groups in both time points compared to healthy animals before operation (p < 0.001). No significant difference between laser and control group. (**F**) Increase of range in ankle angle in laser treated group compared to healthy animals before operation and control group in both time points. The values are expressed as mean ± SEM. n = 5 per group (3 video records of each animal), *p < 0.05, **p < 0.01, ***p < 0.001. BO – before operation, w – weeks after SCI.

A quantitative analysis of the behavioral effects of laser treatment after SCI was carried out by MotoRater (TSE). Locomotor parameters were assessed during over-ground walking, in the 5^th^ and 9^th^ weeks after SCI. The lesion of the spinal cord resulted in a severe impairment of locomotion which did not recover by 9 weeks post surgery. Rats from the non-treated group dragged their bodies over the runway mainly using their forelimbs, without the hindlimb weight support, which was reflected by a significant decrease of the height of the iliac crest (p < 0.001). The laser group also showed a reduced height of the iliac crest in both time points compared to the healthy animals (p < 0.001). However, the laser treated animals regained the body weight supported steps and the group lifted their bodies significantly higher than the controls in both time points (Fig. 2A, 5^th^ week p < 0.05, 9^th^ week p < 0.01).

The kinematic evaluation included calculation of the maximal positive and negative excursion of hindlimbs (Fig. 2B,C). There were no significant changes between the groups in negative excursion (retraction). In contrast to the retraction, the positive excursion (protraction) changed distinctly. The average protraction distance in the healthy animals was +1.7 cm; however the animals were not able to reach positive values after SCI. The laser treated group was significantly closer to zero (p < 0.001).

We further evaluated the range of motion in hip, knee and ankle joints (Fig. 2D–F). We found a significant decrease in the range of movement in a hip joint 5 weeks after the injury in the control group, compared to the uninjured animals (p < 0.05). The range of motion was also significantly decreased in the control group at 5 and 9 weeks compared to the laser group, in both time intervals (p < 0.05). There were no differences between the control and laser groups in knee and ankle in both time intervals. The range of motion was significantly decreased (p < 0.001) in all groups after injury, compared to the healthy animals before the surgery. In the ankle angle, there were no significant differences between the healthy animals and the animals in the control group. We found differences between the healthy rats and the laser treated group (p < 0.001), as well as between the control and laser treated groups (p < 0.001).

### The soleus muscle weight and bone strength

**Figure 3:**
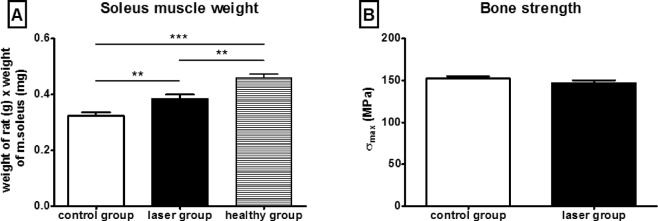
Effect of laser treatment on soleus muscle weight and bone strength. (**A**) Soleus muscles were dissected and weighed immediately after the perfusion. The weight of soleus muscle of laser-treated animals was significantly higher compared to control animals. (**B**) The three-point bending test was used to evaluate mechanical properties of rat femurs. There were no significant differences between groups. The values are expressed as mean ± SEM. Control group n = 11, laser-treated group n = 15, healthy group n = 8. *p < 0.05.

The soleus muscle mass was weighed after transcardial perfusion at 9 weeks after the SCI and the values were expressed relative to the body weight of the animals (Fig. 3A). The laser-treated group had a significantly higher weight of soleus muscles compared to the control group (p < 0.01). The healthy animals had a significantly higher weight of soleus muscles, compared to both groups with SCI (healthy rats versus control group p < 0.001, healthy rats versus laser group p < 0.01). The mechanical properties of rat femurs were evaluated with the use of a three-point bending test. The mean bending strength values for the laser-treated femurs were approx. 3.5% lower than the bending strength values for the control femurs, and this difference was not statistically significant (Fig. 3B).

### Gray and white matter

**Figure 4:**
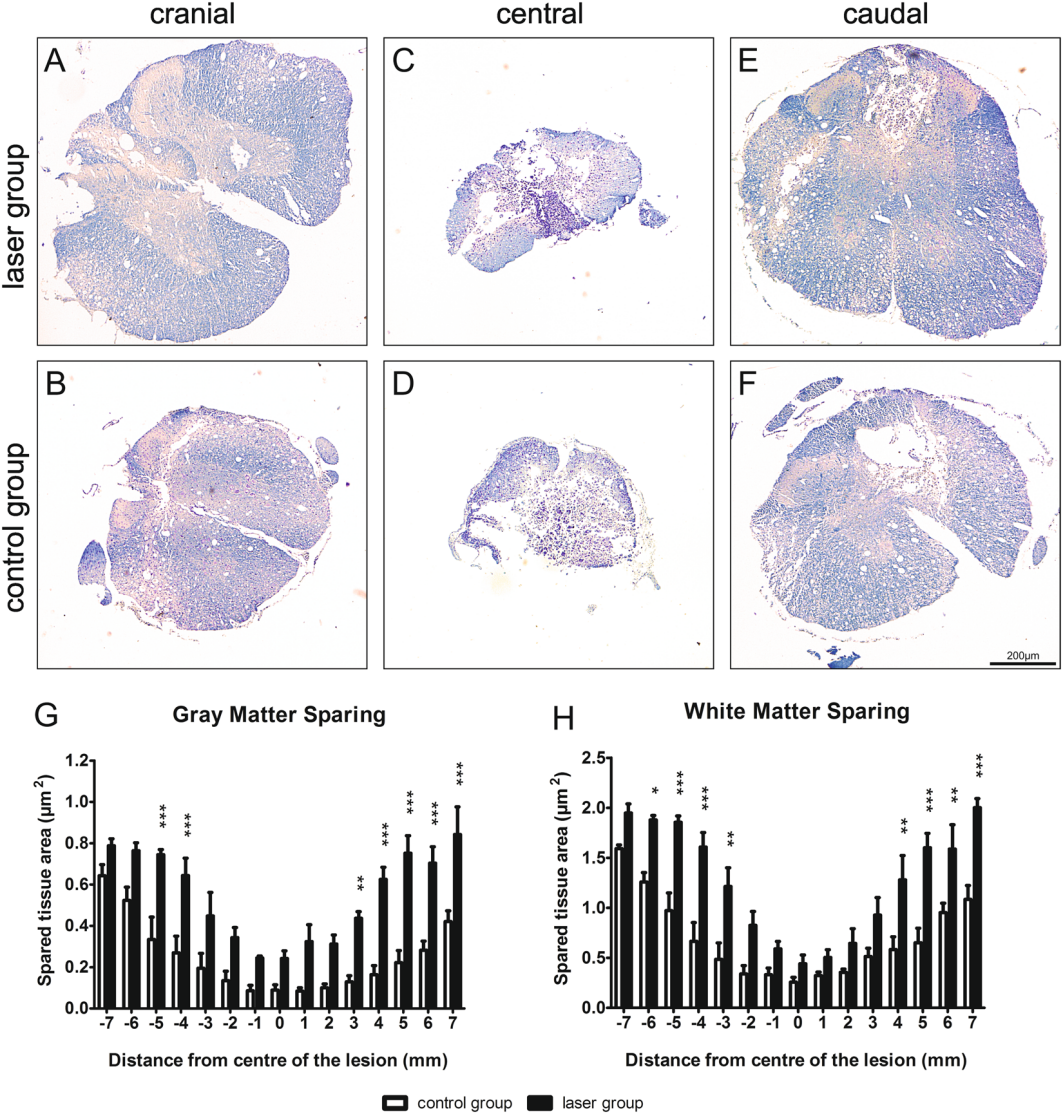
Histological analysis. (**A**,**B**) Representative images of Luxol-Fast Blue and Cresyl Violet stained cross sections 9 weeks after the SCI. (**A**) – laser group, (**B**) – control group. (**C**) Morphometric measurement of gray matter sparing showed a preservation of the tissue throughout the whole analyzed part of the spinal cord with significance 4–5 mm cranially and 3–7 mm caudally from the epicenter. (**D**) White matter was also more spared with significance 3–6 mm cranially and 4–7 mm caudally from the lesion center. The values are expressed as mean ± SEM. n = 6 per group. *p < 0.05, **p < 0.01, ***p < 0.001.

We evaluated the gray and white matter sparing using Luxol-Fast Blue and Cresyl Violet staining and found both to be better preserved in the laser group, in the cranial and caudal parts of the lesion. The gray matter of the spinal cords of the laser-treated rats was significantly more spared, 4–5 mm cranially (p < 0.001) and 3–7 mm caudally (3 mm p < 0.01, 4–7 mm p < 0.001) from the lesion center (Fig. 4A). The laser treatment caused a significant preservation of white matter, 3–6 mm cranially (3 mm p < 0.01, 4–5 mm p < 0.001, 6 mm p < 0.05) and 4–7 mm caudally (4 and 6 mm p < 0.01, 5 and 7 mm p < 0.001) from the epicenter (Fig. 4B).

### Glial scar

**Figure 5:**
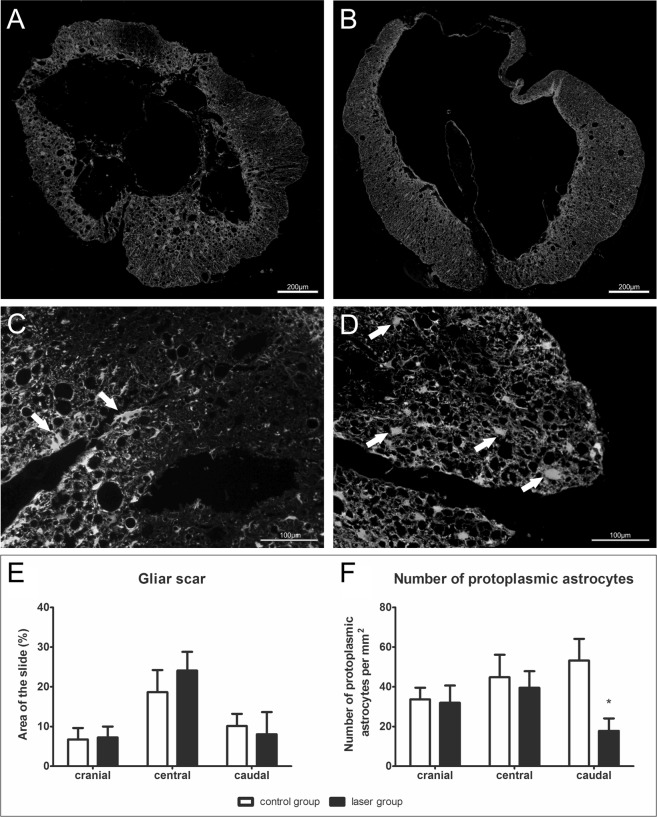
Glial scar formation. (**A**–**D**) Representative images of GFAP staining of cross sections 9 weeks after the SCI for evaluation of glial scar in the cranial part (**A**,**B**) and the number of protoplasmic astrocytes in the caudal part (**C**,**D**). White arrows show the protoplasmic astrocytes. (**A**,**C**) – laser group, (**B**,**D**) – control group.(**E**) Glial scar evaluation in the perilesional area of the spinal cord. Glial scar formation was similar in both groups in cranial (−3 mm, −2 mm), central (−1–+1 mm) and caudal (+2 mm, +3 mm) parts of the lesion center. (**F**) The number of protoplasmic astrocytes was significantly lower in laser-treated rats caudally (+2 mm, +3 mm) from the lesion center. The values are expressed as mean ± SEM. n = 6 per group. *p < 0.05.

The evaluation of astrogliosis was carried out on GFAP stained spinal cord transversal sections in the cranial (−3 mm, −2 mm), central (−1–+1 mm) and caudal (+2 mm, +3 mm) parts of the lesion center. We did not find any statistically significant difference between the groups (Fig. 5A,B,E). We also counted protoplasmic astrocytes and expressed the values as a number of cells/mm^2^. We did not find any differences between the examined groups in the cranial and central parts of the spinal cord lesion. There is a significant decrease of protoplasmic astrocytes (p < 0.05) in the caudal part in the laser-treated group (Fig. 5C,D,F).

### Axonal sprouting

**Figure 6:**
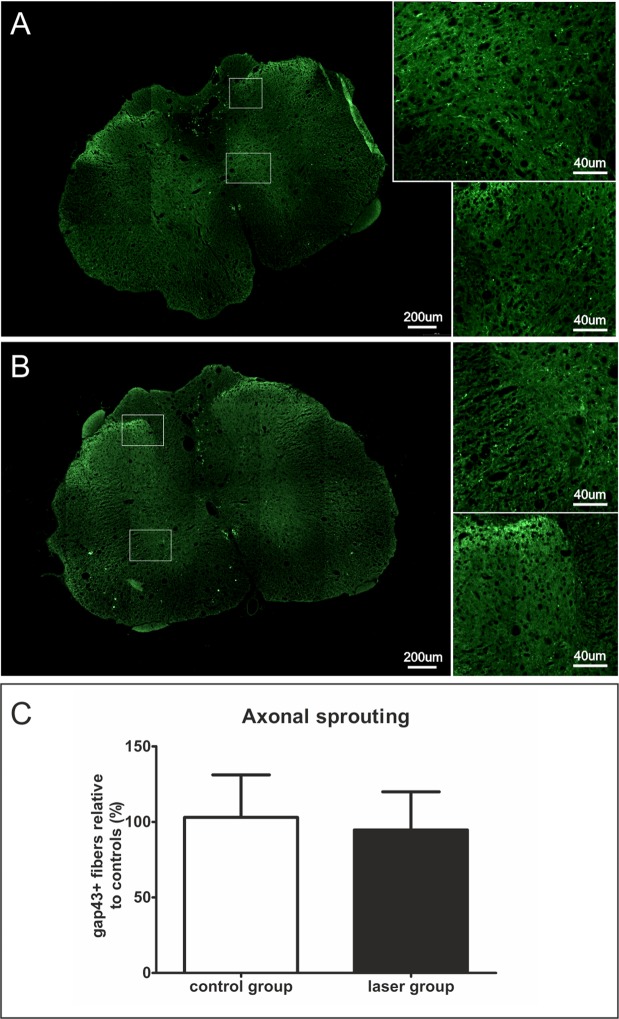
Axonal sprouting. (**A**,**B**) Representative images of GAP43 stained transversal sections 9 weeks after the SCI in the most cranial part of the spinal cord. (**A**) – laser group, (**B**) – control group. (**C**) GAP43 protein expression was similar in both groups. n = 6 per group. The values are expressed as mean ± SEM.

GAP43-staining was used to determine newly sprouted axonal fibers. We did not observe any significant difference in the laser-treated group in comparison with the control group (Fig. 6).

### Microglial/macrophage activation

**Figure 7:**
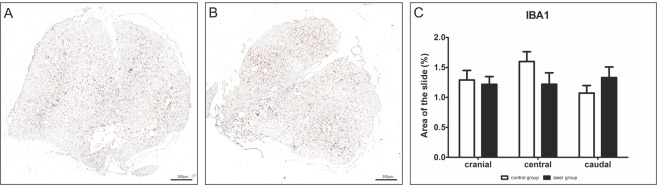
Effect of PBM on microglia/macrophages using IBA1 staining. (**A**,**B**) Representative images of IBA1- stained cross sections 9 weeks after the SCI at the caudal part of the injury site. (**A**) – laser group, (**B**) – control group. (**C**) We found no significant difference between the laser-treated and control groups in cranial (−3 mm, −2 mm), central (−1–+1 mm) and caudal (+2 mm, +3 mm) parts of the lesion center. n = 6 per group. The values are expressed as mean ± SEM.

An immunohistochemical analysis of microglial/macrophage activation was carried out using IBA1 (ionized calcium-binding adapter molecule-1) marker. We did not observe any significant differences in the positive area between the laser-treated and control groups (Fig. 7).

### Analysis of microglia/macrophage polarization

**Figure 8:**
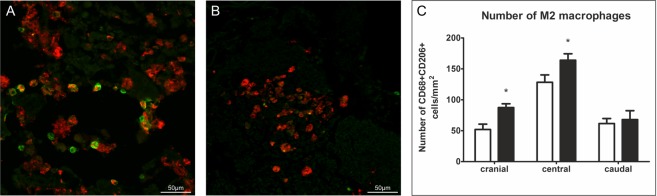
Analysis of microglia/macrophage polarization at a lesion site. (**A**,**B**) The representative images of CD206 (green) and CD68 (red) staining in the central part of the lesion. (**A**) – laser group, (**B**) – control group.(**C**) We considered the double labeled cells to be M2 microglia/macrophages and we found statistically significant differences in their numbers between the group in cranial (−2 mm) and central (epicenter) parts of the spinal cord. There was no difference in the caudal (+2 mm) part. n = 4 per group. The values are expressed as mean ± SEM. *p < 0.05.

The evaluation was carried out using CD68 (red) and CD206 (green) stained spinal cord transversal sections in the cranial (−2 mm), central and caudal (+2 mm) parts of the lesion. A CD68 marker was used to stain microglia/macrophages and CD206 to stain microglia/macrophages polarized to M2 phenotype. We evaluated the number of double labeled cells and the values are expressed as the number of double positive CD68+/CD206+ cells/mm^2^. We found a statistically significant increase of double labeled cells in the cranial and central parts in the laser group (Fig. 8, p < 0.05).

### Analysis of apoptotic cells

**Figure 9:**
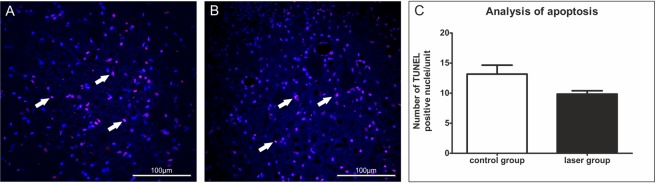
Analysis of apoptotic cells using TUNEL staining. (**A**,**B**) The representative images of TUNEL staining in the caudal part of the lesion. White arrows show the TUNEL and DAPI labeled nuclei. (**A**) – laser group, (**B**) – control group. (**C**) We observed a trend in the apoptosis decrease after laser treatment but it was not statistically significant. n = 4 per group. The values are expressed as mean ± SEM.

An immunohistochemical analysis of apoptotic cells was performed using terminal deoxynucleotidyltransferase (TdT)-mediated dUTP biotin nick-end labeling (TUNEL) assay. We counted cell nuclei double positive for DAPI and TUNEL (Fig. 9). We observed a trend in the apoptosis decrease after laser treatment but it was not statistically significant.

### Quantitative real-time reverse transcription polymerase chain reaction

**Figure 10:**
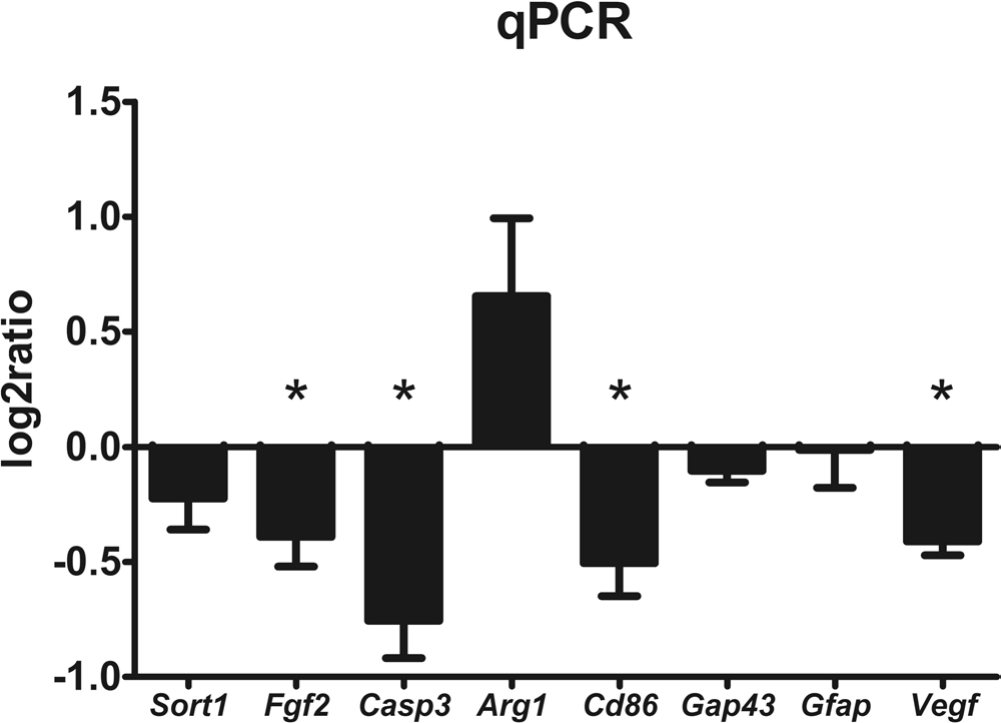
mRNA expression profiling in the lesion center 9 weeks after SCI. The graph shows the log2-fold changes in gene expression in the laser-treated group compared to control animals. Expression of *Fgf2*, *Casp3*, *Cd86* and *Vegf* was significantly downregulated in the spinal cords of laser-treated animals. n = 4 per group. The values are expressed as mean ± SEM. *p < 0.05.

The expressions of genes related to growth factors (*Sort1*, *Fgf2*), immune response (*Arg1*, *Cd86*), axonal sprouting (*Gap43*), astrogliosis (*Gfap*) and vascularization (*Vegf*) were determined 9 weeks after the injury (Fig. 10) using Quantitative real-time reverse transcription polymerase chain reaction (qRT-PCR). We also evaluated the expression of (*casp3*), which can be related to apoptosis, or can also be upregulated in reactive astrocytes. All of the investigated genes were downregulated, except *Arg1* which was upregulated but did not reach the significant level. The decreased expression of *Fgf2*, *Casp3*, *CD86* and *Vegf* was significant compared to the control group (p < 0.05).

## Discussion

Spinal cord injury is a severe traumatic medical condition without any effective treatment to date. Finding a suitable treatment or therapy which could lessen the effects of primary and secondary injury is a big challenge within the neuroscience research field.

In this study, we evaluated the effect of PBM on recovery after spinal cord injury. We used the commercially available MLS laser. The MLS impulse is based on two combined and synchronized emissions: 808 nm continuous and 905 nm pulsed light. This combination should transfer light energy to anatomic structures in a more effective way and modulate cellular metabolism, blood flow and improve the supply of energy.

As an injury model, we chose balloon compression lesion because it is a simple and reproducible model which is clinically relevant to human SCI. The spinal dura remains intact and there is no laminectomy at the site of injury as in other models, which acts as a decompression and possibly controls the secondary phase of injury^38^.

It should be possible for both of our wavelengths to penetrate all the tissue layers surrounding the spinal cord, according to the results of Byrnes *et al*.^33^. Their study showed that 6% of the incident power of 810 nm laser light can penetrate the spinal cord. Our data demonstrated that PBM improved functional recovery after SCI. The results from the BBB test showed that the laser-treated rats performed significantly better the first week following injury. Other studies also showed an increased BBB score after laser treatment^33,35,39^. The BBB test is a useful tool to evaluate the general overall locomotor ability but does not reflect specific changes in motor function^40^. Therefore, we used the beam walk test to give a more appropriate indication as to the specific abilities (paw placing, strength and agility). The laser-treated animals were able to cross the beam in a shorter time and obtained a higher score in this test. Byrnes *et al*.^33^ applied the ladder beam test which also requires more skilled coordination. They observed a decrease in ladder cross time 9 weeks after the T9 dorsal hemisection. The quantification of the locomotor activity of the hindlimb confirmed the improvement of the locomotion recovery. In the parameter height of the iliac crest, the protraction of the hindlimb and angle range of the hip, the laser–treated animals were nearer to those of the uninjured animals. Surprisingly, the angle range of the ankle was significantly increased in the laser-treated animals compared to the control and healthy animals. The overuse of the ankle could be caused by the compensatory adaptation of movements to allow effective locomotion.

Our results from the plantar test, demonstrating the decrease of post-injury hypersensitivity in laser-treated animals, are in accordance with other findings. Hu *et al*.^41^ also reported the effect of light on hypersensitivity after mild T10 hemicontusion injury using 670 nm LED light. Veronez *et al*.^42^ observed an improved tactile sensitivity after 1000 J/cm^2^ irradiation of the spinal cord, contused between T9 and T10 level.

SCI is usually accompanied by muscle atrophy caused by decreased muscle activity. There is evidence that PBM can increase biochemical activity and improve morphological recovery in muscles after peripheral nerve injury^43,44^. In our study, the weight of the soleus muscle of the laser-treated animals was increased compared to the control group. These results suggest that PBM slowed down the atrophic processes in muscles after SCI. We did not analyze the neuromuscular junction denervation, due to the previous results of neuromuscular junction degeneration and remodeling being inconclusive; indicating a heterogeneous response to injury^45^.

We investigated the effects of PBM on bone strength after SCI. Shortly after the injury there are bone remodeling processes leading to osteoporosis. Medalha *et al*.^46^ found PMB to be effective on the healing of bone defects in rats after the complete transection of the spinal cord. However when we investigated the mechanical properties of uninjured tibia bones, we did not find any differences between the treated and untreated groups. This could be due to the short time period after SCI (9 weeks), so the osteoporosis may not yet have developed, or that the testing apparatus was not able to detect subtle changes in the bone remodeling between the tested groups.

The histomorphometric findings correlate with the observed motor and sensory recovery. We speculate that the major effect of PBM is due to its neuroprotective effect, as in the laser-treated group we found a significantly greater area of preserved gray and white matter in the peripheral sites of the injury. Similarly, Paula *et al*.^39^ also observed a greater area of preserved nervous tissue on day 20 post-op, in the laser-treated animals after spinal cord injury, which was caused by dropping 10-g of weight. Wong-Riley *et al*.^47^ confirmed these findings when an *in vitro* study established that LED treatment can reduce the death of functionally inactivated primary neurons by restoring the function of mitochondrial enzyme cytochrome c oxidase.

Byrnes *et al*.^33^ demonstrated that light with 810 nm wavelength, at a dosage of 1 589 J/cm^2^ improved axonal regrowth after dorsal hemisection. Wu *et al*.^35^ also showed improved regrowth of axons with the same laser structure after hemisected and contused spinal cord, using anterograde labeling. We did not confirm the hypothesis that PBM should influence the axonal sprouting, owing to the fact that we did not find any difference in the number of GAP43+ fibers compared to the laser and control groups. Our qRT-PCR results also did not show any increase of *gap43* mRNA transcript 9 weeks after the injury.

We did not observe any effect of the treatment on glial scar formation using immunostaining and gene expression of GFAP. However, the number of protoplasmic astrocytes was reduced caudally from the lesion epicenter. The protective effect of the light therapy on the caudal part of the lesion was also suggested by Song *et al*.^48^. Additionally, they found an increased number of anti-inflammatory M2 microglia/macrophages. Von Leden *et al*.^49^ investigated the polarization of the microglia/macrophages in different energy densities. They found that the energy between 0.2–10 J/cm^2^ can induce polarization of microglia/macrophages into M2 phenotype. Our data suggests the same trend of a polarization shift towards the M2 microglia/macrophages by a significant mRNA downregulation of Cd86 (marker of inflammatory M1) and a slight upregulation of Arg1 (marker of M2). The overall activation of microglia/macrophages is similar in the laser-treated and control groups according to the immunohistological evaluation. The number of double labeled CD206+/CD68+ cells (counted as M2 microglia/macrophages) was significantly increased in the cranial and central parts of the lesion, supporting the effect of PBM on the microglia/macrophage polarization. However, a deeper insight into macrophage/microglia polarization would require further analysis of additional M1 and M2 markers.

We found a downregulation of *Sort1*, *Fgf2*, *Casp3* and *Vegf* genes. Sortilin is a membrane receptor expressed in neuronal tissue which binds the nerve growth factor (NGF) and its precursor proNGF. These neurotrophines regulate neuronal development through cell survival and cell death signaling. Sortilin interaction with the p75 neurotrophin receptor has been demonstrated and this subsequently induces apoptosis after the binding of proNGF^50^. Neuronal cells then secrete the precursor proNGP which induces apoptosis by binding to the sortilin receptor^51^. We discovered that although the level of mRNA of *Sort1* gene downregulated after the laser treatment, it did not reach the significance level. NGF is also involved in hyperalgesia development^52^. The downregulation of S*ort1* consequently has a connection with the reduction of thermal hypersensitivity in the laser treated animals.

*The Casp3* gene can be either associated with apoptosis, or it can be upregulated in reactive astrocytes without cell death^53^. Our labeling with TUNEL did not confirm a significant decrease in cell apoptosis 9 weeks after SCI in laser treated animals. Alternatively, the *casp3* downregulation may highlight the observed reduction of protoplasmic astrocytes after PBM therapy.

The level of vascular endothelial growth factor (VEGF) is reduced after SCI and a decrease of VEGF lasts up to 1 month after SCI^54^. The neutralization of VEGF with antibody neither attenuated nor exacerbated chronic histopathology or functional recovery and the role of VEGF in the angiogenesis after SCI is limited^55^. The downregulation of vascular endothelial growth factor may be connected to the described decrease of hypersensitivity after SCI as suggested by the results of Nesic *et al*.^56^. VEGF-A isoform 165 was found to induce hypersensitivity and mechanical allodynia in rats after SCI.

Yoshida *et al*.^57^ demonstrated that the intrathecal application of anti-fibroblast growth factor-2 (FGF-2) antibodies, in a rat model of neuropathic pain, suppresses the increase of FGF-2 and GFAP positive cells and significantly attenuated the mechanical allodynia in rats. In this study, we found that treatment with PBM suppresses the gene expression of *Fgf-2*and this could be connected with the significant reduction of hyperalgesia. We further discovered a reduction in the number of protoplasmic astrocytes in the caudal part of the spinal cord injury.

PBM is a non-invasive therapeutic intervention. The intensity and frequency of light is clearly an important factor. However, the range of the light sources used in published studies varies from 6 J/cm^2^ ^39^ to 1,500 J/cm^2^ ^58^ making it difficult to correlate our results with other studies in terms of intensity and frequency, or the energy of different light sources. Our study was focused on the long term effects and functional recovery after PBM application. Further biochemical analyses, immediately after light application, will assist further studies to resolve all mechanisms leading to positive changes after laser treatment. However, we have demonstrated the positive effect of the combination of continuous and pulsed wave lights of a commercially available laser device on regeneration after acute SCI. These results suggest that light is a promising therapy for human SCI.

## Methods

### Animals and ethics statement

Adult male Wistar rats (n = 26, 300 ± 15 g) were housed in pairs and maintained at 22 °C and on a 12 h light/dark cycle. Food and water were provided *ad libitum*. All experiments were performed in accordance with the European Communities Council Directive of 22nd of September 2010 (2010/63/EU) regarding the use of animals in research, and were approved by the Ethics Committee of the Institute of Experimental Medicine ASCR, Prague, Czech Republic.

### Spinal cord injury

A balloon-induced compression lesion was performed as described previously^38^. Briefly, rats were anesthetized with 2–3% isoflurane (Forane, Abbott Laboratiores, UK). The skin was incised on the dorsomedian line from Th8 to Th12. The soft tissue and spinous processes of vertebrae Th10 and Th11 were removed. A 2-french Fogarty catheter (Baxter, Irvine, USA) was placed cranially in the epidural space through laminectomy at Th10 vertebrae. The catheter was positioned at the Th8 spinal level. Spinal compression was created by fast balloon inflation with 15 µl of saline for 5 minutes. The catheter was then quickly deflated and removed. The muscles and skin were sutured in anatomical layers. The animals received gentamicin sulfate (Lek Pharmaceutical, Slovenia, 5 mg/kg) for 5 days to prevent infections and carprofen (Rimadyl, Pfizer, USA, 7,5 mg/kg) to reduce postoperative pain. Postoperative care included manual bladder expression twice per day.

### Laser treatment

Treatments were performed with a MLS laser (Mphi laser, ASA Srl, Italy). MLS laser is a NIR laser with two synchronized sources (laser diodes). The first one is a pulsed laser diode, emitting at 905 nm, with 25 W peak optical power and frequency of the pulse 10 Hz. The second laser diode (808 nm) was operated in a continuous mode with power 1 W. Both of the laser beams were synchronized.

After the SCI, the animals were randomly divided into two experimental groups. The first group (laser-treated group, n = 15) received laser treatment for 10 consecutive days starting 15 minutes after the injury. The irradiation was done at one area (3 cm^2^) located 3 cm above the place of injury. The point was treated for 9 min and 5 sec with the following parameters: 10 Hz, CPW, 100% intensity, 299.946 J/total. The control group (n = 11) only underwent general anesthesia with 2% isoflurane for 9 min and 5 sec.

### Behavioral assessment

#### BBB test

The Basso, Beattie, and Bresnahan test^59^ was used to assess locomotor activity after SCI. The BBB scale ranges from 0–21 and reflects the extent of hindlimb joint movement, weight support, toe clearance, paw placement and forelimb-hindlimb coordination. Each hindlimb is scored separately and then averaged for the analysis. The test was performed weekly from the first week after the injury.

#### Plantar test

The hind paw sensitivity to thermal stimulus was tested using Ugo Basile test (Ugo Basile, Italy). The animal was situated into a clear box and the source of infrared heat was focused on the central part of the hind paw. The latency of the hindpaw withdrawal response was assessed five times for each paw and the highest and lowest values were excluded from the final analysis. The test was performed one week before SCI and then weekly. The significant decrease of withdrawal latency was defined as hyperalgesia.

#### Beam walk test

The recovery of motor function and fore-hindlimb coordination was appraised by the beam walk test. The task is to cross a 140 cm long rectangular wooden beam (width 3 cm) to enter the dark box placed at the end of the beam. The latency and walking distance was measured in 60 seconds in the central part of the beam by a video tracking system (TSE-Systems Inc., Germany) and the limb movement and coordination were evaluated using a 7-point scale modified from Goldstein^60^. The rats were pre-tested one week before surgery and then tested from the third week. The test was performed twice per day by each animal for 3 consecutive days per week.

### Locomotor quantification

A kinematic analysis of locomotor performance was conducted using MotoRater apparatus (TSE Systems, Germany) as previously described^61^. In brief, the rats were trained to cross an illuminated glass-walled corridor to reach the dark escape box at the end. The performance was recorded from the bottom by a mobile high-speed camera at 200 frames per second. Two side mirrors enable the recording of the animals simultaneously from three sides. Prior to testing, the skin overlying the hindlimb joints - iliac crest, hip, knee, ankle, 5th metatarsophalangeal (MTP) joint - was tattooed by black ink with a tattoo set (Harvard Apparatus, UK). Training was performed one week before the surgery and the testing was done in the 5^th^ and 9^th^ weeks after the SCI. Four to six runs were recorded and three representative runs were analyzed per animal using automatized TSE Motion software (TSE Systems, Germany). The first and the last steps in a run were excluded. The parameters for analysis were defined as follows: height of iliac crest as a maximal vertical distance between the iliac crest and the runway, protraction and retraction of the hindlimb as a maximal positive (protraction) or negative (retraction) distance of the MTP joint relative to the iliac rest. We measured a range of angles in the hip, knee and ankle joints of both hindlimbs. The values were determined for each limb and then averaged for the analysis.

### Tissue processing

For histological analysis, 9 weeks after the SCI rats were transcardially perfused with ice cold 4% paraformaldehyde (Penta s.r.o., Czech Republic) in phosphate-buffered saline, the spinal cords were postfixed overnight at 4 °C and afterwards carefully extracted from the vertebral column. A 3-cm long segment with the epicenter in the middle was paraffin embedded and transversely cut. Immediately after the perfusion, soleus muscle of both hindlimbs was extracted and weighted.

### Bone strength measurement

A three-point bending test was used to measure the mechanical properties of rat femurs. The femurs were removed and were cleaned of tissue, and they were then immediately soft wrapped in gauze soaked in isotonic saline and frozen for preservation. After thawing, the bones were hydrated in a physiological saline solution at room temperature (23 °C ± 5 °C) for a period of 24 h before the experiment. Prior to mechanical testing, all soft tissues were removed and the specimens were placed on two rounded bars set 15 mm apart, in order to apply the load from the medial side of the rat femur^62,63^. The MTS Mini Bionix 858.02 biomechanical servohydraulic testing system (MTS, Minnesota, USA) was used for the three-point bending test. The bending strength σ_omax_ (MPa = N.mm^−2^) was calculated from the ultimate force F_max_ (N) and the section modulus of the bone W_omin_ (mm^3^). The ultimate load (the force at failure) is the maximum load that a specimen withstands before fracture. The bones were measured according to the following principle: the bone cross-section at the location of the fracture was resurfaced by grinding, in order to obtain a planar surface perpendicular to the axis of the femur. The cross-section surface was marked with a colored felt-tip pen and was scanned by a flat tabletop scanner at a resolution of 1200 × 1200 dpi, i.e. at a pixel size of 0.021 × 0.021 mm. The full-color digital image obtained from the scanner was mathematically processed and the important dimensions of the bone cross-section were assessed. Finally, the strength was calculated as an intrinsic property of the bone. Load-deflection curves were recorded at the cross-head of 2.0 mm/min by a load cell with a measuring range of 0–500 N (detector error max. 0.05%, e.g. 0.08 N error when 160 N is applied). The mechanical tests were also carried out at room temperature (23 °C ± 5 °C).

### Histological analysis

Serial cross sections (thickness 5 µm) were stained with Luxol-Fast Blue and Cresyl Violet (Sigma-Aldrich, USA) to distinguish the white and gray matter. Images of each cross-section were taken with an Axioskop2 plus microscope (Carl Zeiss AG, Germany) and analyzed by ImageJ software (National Institutes of Health, USA). For morphometric measurements, 15 sections were chosen at 1 mm intervals along the cranio-caudal axis. The area of preserved tissue (mm^2^) was calculated on each section. The epicenter was defined as the smallest area of the preserved spinal cord.

### Immunohistochemical analysis

Paraffin mounted cross sections were used for immunohistochemical staining. To evaluate axonal sprouting, primary antibody GAP43 (1:2000, Millipore, USA) was used, combined with a secondary antibody conjugated with Alexa-Fluor 488 (1:200, Abcam, UK). Microscope LEICA CTR6500 (TissueGnostics, Austria) was used to acquire high magnification images for analysis of Gap43 positive fibers which was conducted using TissueQuest software (TissueGnostics, Austria).

For the analysis of astrogliosis, anti-GFAP antibody conjugated with CY3 1:400 was used (Sigma Aldrich, USA). For the analysis of microglial/macrophages activation, sections were incubated with goat anti-IBA1 antibody (1:500 (Abcam, UK) followed by biotinylated rabbit anti-goat IgG and then streptavidin-peroxidase (Vector Laboratories, USA). Finally, the sections were reacted in a 3,3′-Diaminobenzidine (Vector Laboratories, USA) and stained for hematoxilin. The evaluation of microglia/macrophage polarization was carried out using CD68 (1:75, Bio-Rad, USA), combined with a secondary antibody conjugated with Alexa-Fluor 594 (1:500, Abcam, UK) and CD206 (1:200, Abcam, UK) also combined with a secondary antibody conjugated with Alexa-Fluor 488 (1:200, Abcam, UK). To detect apoptotic cells, the terminal deoxynucleotidyltransferase (TdT)-mediated dUTP biotin nick-end labeling (TUNEL) assay (ApopTag® Red *In Situ* Apoptosis Detection Kit, Millipore, USA) was used according to the manufacturer’s instructions.

Microscope LEICACTR6500 (TissueGnostics, Austria) was used to take images of spinal cross sections. The number of protoplasmic astrocytes and microglial activation was analyzed using ImageJ software. GFAP stained sections were compared to healthy controls and the number of enlarged GFAP-Cy3 positive cell bodies was counted within the close vicinity to the lesion cavity as well as in the surrounding white and gray matter areas. Quantification of the GFAP positive area near the lesion cavity was conducted using TissueQuest analysis software (TissueGnostics, Austria,) in cranial (−3 mm, −2 mm), central (−1–+1 mm) and caudal (+2 mm, +3 mm) around part of the lesion center. Quantification of double positive CD206+/CD68+ cells was carried out using ImageJ software. The double labeled cells were counted and their number to mm^2^ was calculated. The number of apoptotic cells was counted around the lesion cavity (−3–+3 mm) using custom-made script and recalculated the number of positive cells per unit area.

### qRT-PCR

Quantitative RT-PCR was used to evaluate the expression of the genes *Sort1* (Rn01521847_m1), *Fgf2* (Rn00570809_m1), *Casp3* (Rn00563902_m1), *Arg1* (Rn00691090_m1), *Cd86* (Rn00571654_m1), *Gap43* (Rn01474579_m1), *Gfap* (Rn01253033_m1) and *Vegf* (Rn01511601_m1) 9 weeks after the SCI (n = 4 in both groups). The RNA was isolated from paraformaldehyde-fixed spinal cord tissue by the High Pure RNA Paraffin Kit (Roche, Germany) following the manufacturer’s recommendations. Isolated RNA was quantified by a spectrophotometer (NanoPhotometerTM P-Class, Germany). The reverse transcription into cDNA was carried out using Transcriptor Universal cDNA Master (Roche, Germany) and a thermal cycler (T100^TM^ Thermal Cycler, Bio-Rad, USA). The qRT-PCR reactions were done using a cDNA solution, FastStart Universal Probe Master (Roche, Germany) and TaqMan^®^ Gene Expression Assays (Life Technologies, USA). The reaction was performed in a final volume of 10 µl containing 25 ng of RNA. A real-time PCR cycler (StepOnePlus^TM^, Life Technologies, USA) was used to perform the amplification. The cycling conditions were set as follows: 2 min at 50 °C, 10 min at 95 °C, followed by 40 cycles of 15 s at 95 °C and 1 min at 60 °C. Each array contained a negative control and all samples were run in duplicate. The obtained results were analyzed using StepOnePlus^®^ software (Life Technologies, USA). The gene expression was normalized using *Gapdh* (Rn01775763_g1) as a reference gene. The relative quantification of gene expression was determined using the ∆∆*C*t method. Control rats with SCI only were set as zero. A log2 scale was used to express the symmetric magnitude for up and down regulated genes.

### Statistical analysis

The comparison of the laser-treated and control groups was carried out using several statistical tests. For behavioral assessment, gray and white matter sparing, microgliosis, macrophages polarization, glial scar and the number of protoplasmic astrocytes, the two-way repeated measurement ANOVA with Student-Newman-Keuls post-hoc test was applied. For quantitative locomotor analysis, one-way ANOVA with Newman-Keuls Multiple Comparison Test was used. For axonal sprouting, weight of soleus muscle, TUNEL and qPCR analysis, a student unpaired nonparametric T-test with Mann Whitney test was used for evaluation. The data are expressed as arithmetical mean ± SEM. The statistical analysis was carried out using GraphPad Prism5 software (San Diego,USA).

## Data Availability

The datasets generated during and/or analyzed during the current study are available from the corresponding author on reasonable request.
